# Parotid preservation or immune protection? The dual dilemma in head and neck cancer radiotherapy

**DOI:** 10.3389/fimmu.2025.1634522

**Published:** 2025-09-05

**Authors:** Jianing Qin, Fengjuan Zhang, Wenjing Zhang, Song Yang, Bin Yang, Tao Xie, Hui Zhang, Min Wan, Huachi Li

**Affiliations:** ^1^ Department of Oncology, Liuzhou People’s Hospital Affiliated to Guangxi Medical University, Liuzhou, Guangxi, China; ^2^ Department of Radiation Oncology, Hubei Cancer Hospital, Tongji Medical College, Huazhong University of Science and Technology, Wuhan, Hubei, China; ^3^ Department of Thyroid and Breast Surgery, Honghu People’s Hospital of Hubei Province, Jingzhou, Hubei, China; ^4^ Department of Oncology, Hubei Cancer Hospital, Tongji Medical College, Huazhong University of Science and Technology, Wuhan, Hubei, China; ^5^ Department of Gynaecology, Yangxin County Maternity and Child Health Hospital/Yangxin County Children’s Hospital, Huangshi, Hubei, China; ^6^ Department of Gastrointestinal Surgery, Hubei Cancer Hospital, Tongji Medical College, Huazhong University of Science and Technology, Hubei Provincial Clinical Research Center for Colorectal Cancer; Wuhan Clinical Research Center for Colorectal Cancer, Wuhan, Hubei, China

**Keywords:** head and neck cancer, radiotherapy, radiation injury, parotid gland, immune microenvironment

## Abstract

Head and neck cancer (HNC) represent a common category of malignant tumors. Radiotherapy, as the primary treatment modality for these tumors, while effectively controlling tumor progression, is often accompanied by a series of treatment-related side effects. As a major salivary gland in the head-and-neck region, the parotid gland (PG) is particularly susceptible to radiation damage during radiotherapy, given its anatomical proximity to the target irradiation area. The radiation dose and irradiated volume of the PG not only disrupt its physiological secretory function, leading to debilitating side effects like xerostomia and dysphagia, but also potentially compromise tumor control and patient outcomes by modulating the local and systemic immune homeostasis. This article systematically reviews the relevant research on the impact of PG irradiation on the immune microenvironment during HNC radiotherapy in recent years, and it delves into multiple levels, including cytokine changes and immune cell function alterations, aiming to offer a comprehensive theoretical basis and novel research perspectives for optimizing radiotherapy plans, reducing radiotherapy-related adverse reactions, and improving patient prognosis.

## 1 Introduction

Head and neck cancer (HNC) encompass a wide-ranging and complex group of malignancies, including oral cancer, oropharyngeal cancer, nasopharyngeal cancer, laryngeal cancer, hypopharyngeal cancer, thyroid cancer, and salivary gland cancer. These tumors originate from the intricate and vital anatomical structures within the head and neck region, which houses numerous critical organs and tissues (1). Radiotherapy, due to its unique advantages, occupies a central position in the comprehensive treatment system for HNC (2). For patients with HNC, radiotherapy can be administered as a single therapeutic modality, or combined with chemotherapy, immunotherapy, or novel radiotherapy techniques (3). By precisely targeting tumor tissue, it effectively inhibits tumor cell proliferation, controls lesion progression, and ultimately improves patient survival rates (4).

However, the double-edged sword nature of radiotherapy can’t be ignored (5). In the process of killing tumor cells with radiation, it is inevitable to damage the surrounding normal tissues, and the parotid gland (PG) is one of the sensitive organs that is highly susceptible to damage (6). After impaired PG function, patients may experience significant symptoms such as xerostomia, greatly reducing their quality of life (QOL) (7). In the radiotherapy of HNC, the PG is frequently irradiated due to its anatomical location adjacent to the target area, and its immune damage mechanism exhibits universality: damage to the salivary gland parenchyma leads to a decrease in immunologically active substances and disruption of the local immune cell network, thereby triggering defects in oral immune defense (8). Clinical data have shown that regardless of whether the primary site is the nasopharynx, oropharynx, or larynx, when the irradiation dose to the PG exceeds 40 Gy, the level of salivary IgA decreases by 40%-60%, and the risk of oral infection increases in a dose-dependent manner (9, 10).

Over the past decade, an extensive body of clinical investigations and pre-clinical research endeavors have elucidated that radiation exposure to the PG not only elicits localized tissue injury but also exerts far-reaching effects on the systemic immune microenvironment via intricate biological pathways (9).

The immune microenvironment, a pivotal determinant governing tumor biological dynamics, constitutes a complex ecosystem of immune cells, cytokines, and extracellular matrix components. This intricate network orchestrates a series of critical processes, including tumor initiation, progression, invasive behavior, metastatic dissemination, and therapeutic responsiveness (11). Therefore, exploring the specific impact mechanism of PG radiation dose and volume parameters on the immune microenvironment in HNC radiotherapy has significant clinical guidance value and scientific exploration significance for optimizing radiotherapy plan design, balancing treatment benefits and toxic side effects, and achieving precise personalized radiotherapy strategies.

## 2 Overview of radiotherapy for HNC

### 2.1 Radiotherapy’s indispensable role and efficacy for HNC

Radiotherapy plays an irreplaceable role in the comprehensive treatment system for HNC. In the field of HNC, radiotherapy can be used either as a single curative treatment or in combination with other therapies such as chemotherapy, surgery, immunotherapy (12). For early HNC, such as early nasopharyngeal carcinoma (NPC), laryngeal cancer, radiotherapy alone can often achieve local control of the tumor, achieve treatment effects similar to surgery, and maximize the preservation of the functional integrity of head and neck organs in patients, including swallowing, vocalization, and so on, greatly improving the patient’s QOL (13, 14). For patients with advanced HNC, the concurrent chemoradiotherapy (CCRT) has significantly improved local control rates and survival rates (15). In addition, radiotherapy is also crucial in postoperative adjuvant therapy, as it can reduce the risk of local tumor recurrence and consolidate the effectiveness of surgical treatment (16).

In recent years, with the rapid development of radiotherapy technology, such as intensity-modulated radiotherapy (IMRT), image-guided radiotherapy (IGRT), stereotactic radiotherapy (SBRT), and proton heavy ion radiotherapy, the accuracy of radiotherapy has been significantly improved. While more effectively killing tumor cells, it can better protect surrounding normal tissues and organs, reduce radiotherapy-related toxicities and side effects, and further highlight the advantages and importance of radiotherapy in the treatment of HNC (17, 18).

### 2.2 Common techniques for radiotherapy of HNC

Radiotherapy is an essential component in the treatment of HNC, playing a crucial role in improving patient outcomes. External beam radiotherapy, the most commonly used method, utilizes high-energy X-rays or electrons generated by linear accelerators to irradiate tumors from outside the body. This technique can precisely target the tumor site, delivering a lethal dose of radiation to cancer cells (19). Brachytherapy, on the other hand, involves placing a radioactive source directly into or near the tumor. It is particularly effective for treating tumors in well-defined regions, such as certain oral and cervical cancers. The close proximity of the radiation source to the tumor allows for a high dose of radiation to be delivered locally while minimizing damage to surrounding normal tissues (20).

In HNC radiotherapy, 3D-CRT offers better conformity than 2D-CRT by shaping fields to tumor volume, reducing dose to adjacent normal tissues but exposing larger normal volumes to sub-lethal doses (21). IMRT enables customized dose distributions via intensity modulation, improving tumor control and sparing salivary glands to reduce xerostomia, yet has complex planning and higher scatter radiation risk (21). Volumetric modulated arc therapy (VMAT) shortens treatment time through arc delivery with excellent conformity but demands strict positioning accuracy and quality assurance (22). SBRT provides high local control for small tumors with few fractions but is limited to small lesions and risks late toxicities (23). Proton therapy uses Bragg peaks for precise dose deposition, minimizing normal tissue dose (24).

The efficacy of radiotherapy for HNC varies depending on several factors, including tumor type, stage, and patient characteristics. For early-stage HNC, radiotherapy alone can achieve high cure rates. For example, in early-stage laryngeal cancer, radiotherapy can often preserve laryngeal function while providing comparable survival outcomes to surgery (25). In locally advanced disease, CCRT has become the standard of care, significantly improving both local control and overall survival compared to radiotherapy alone (26). However, despite these advances, some patients still experience recurrence, highlighting the need for continued research to improve treatment outcomes.

While radiotherapy is effective in treating HNC, it is not without side effects. Acute toxicities commonly occur during or shortly after radiotherapy and include skin reactions, mucositis, xerostomia, and dysphagia. Skin reactions can range from mild erythema to severe desquamation, depending on the radiation dose and fractionation schedule. Mucositis, manifested as inflammation and ulceration of the oral and pharyngeal mucosa, can cause significant pain and difficulty in eating and swallowing. Xerostomia, is a common and often persistent side effect resulting from damage to the salivary glands, which can severely impact the patient’s QOL. Dysphagia may also occur due to radiation-induced inflammation and fibrosis of the pharyngeal and esophageal tissues, leading to swallowing difficulties and potential nutritional problems (27).

In addition to acute toxicities, radiotherapy can also cause long-term or late effects. These may include radiation-induced fibrosis, which can affect the function of various organs, such as the larynx, pharynx, and neck muscles, leading to speech and swallowing problems. Radiation-induced brain injury is another potential late complication, which can present as cognitive impairment, memory loss, and neurological deficits. Moreover, there is an increased risk of developing secondary malignancies in the irradiated area over time (28).

### 2.3 Radiation exposure of PGs during radiotherapy

In the implementation of radiotherapy for HNC, based on the target area setting of the radiotherapy plan, some PGs tissues are inevitably included in the irradiation field range (29). Clinical research and imaging monitoring results show that during radiotherapy, the volume of parotid tissue exhibits a dynamic trend of change (30). Research has shown that from the beginning of radiotherapy to the 16th radiotherapy, the contraction amplitude of PGs volume reaches its peak. As the treatment continues, the rate of volume change gradually slows down and tends to stabilize (31). After the radiotherapy course is completed, the average loss rate of PGs volume is estimated to be in the range of 33% (32). Additionally, during the treatment regimen, multiple variables come into play. Inadequate nutritional intake causing weight loss, coupled with metabolic adaptations, and the shrinkage of lymph nodes due to tumor regression, collectively induce positional changes in the PGs. As a consequence, the PGs tend to reposition themselves within high-dose radiation fields, leading to a marked increase in their average radiation exposure (33). The changes in radiation dose and volume reduction of the PGs will have multidimensional effects on its physiological functions such as secretion and digestion, as well as the distribution of local immune cells and cytokine secretion in the immune microenvironment.

## 3 The physiological functions and immune related characteristics of the PGs

### 3.1 Physiological functions of PGs

The PGs, the largest among the major salivary glands in the human anatomy, play a crucial role in maintaining oral homeostasis by secreting saliva, a complex fluid essential for multiple physiological processes (34). Saliva has various important physiological functions, such as moistening the mouth, aiding digestion, cleaning the mouth, and antibacterial properties (35). Saliva produced by the PGs harbors a diverse array of bioactive components, including lysozyme and immunoglobulin A (IgA). These constituents are integral to the establishment and maintenance of oral immune homeostasis, functioning synergistically to protect the oral mucosa against pathogenic invasions and maintain a healthy microenvironment (36).

### 3.2 Characteristics related to PGs and immunity

As a major exocrine gland in the human body, the PGs serves a dual-function capacity within the immune defense framework, contributing both to local mucosal immunity and systemic immunomodulation (37). On the one hand, it continuously secretes saliva rich in immune active substances such as lysozyme, IgA, lactoferrin, etc. through the synergistic effect of acinar cells and ductal cells. These components can directly act on the surface of oral mucosa, and construct the first line of defense against pathogen invasion by inhibiting bacterial adhesion, neutralizing viral activity, regulating microbial balance, and other mechanisms (38). Conversely, the parenchyma of the PGs harbors an innate immune cell network, encompassing lymphocytes such as T and B cells, plasma cells, dendritic cells, and other immune cell subsets. In concert with the extracellular matrix and cytokines, these cellular and acellular components synergistically constitute a highly specialized local immune microenvironment, uniquely tailored to the glandular tissue (39).

However, the ionizing radiation damage suffered by the PGs during radiotherapy for HNC induces immunogenic cell death, promotes inflammation and anti-tumor response, increases the secretion of immunosuppressive antibodies, and depletes immune cells (40). High dose radiation can directly damage the DNA structure of immune cells, leading to cell cycle arrest, apoptosis, or necrosis, while damaging the integrity of the cell membrane and organelle function, making immune cells unable to perform antigen recognition, signal transduction, and immune effector functions normally (41). Radiation-induced structural damage and secretory dysfunction of the PGs can significantly weaken the oral immune defense system, directly increasing the risk of infections. In terms of bacterial infections, after radiotherapy for HNC, the concentrations of antimicrobial substances such as lysozyme and lactoferrin in saliva decrease by 30%-50%. This leads to a significant increase in the colonization rate of opportunistic pathogens such as streptococci and staphylococci in the oral cavity. Clinical data show that the incidence of gingivitis and periodontitis increases from 10%-15% before treatment to 35%-50%, and in severe cases, it can progress to maxillofacial space infection (42). The risk of viral infections is also closely related to the immune function of the PGs. Secretory immunoglobulin A (sIgA) in saliva is the core substance against oral viral invasion. After radiotherapy, the secretion of sIgA from the PGs decreases by 40%-60%, increasing the risk of herpes virus (such as HSV-1) infection by 2 – 3 times, with an incidence rate of 15%-20%. It manifests as oral mucosal herpes and ulcers, prolonging the mucosal repair cycle (43). In fungal infections, the reduced salivary flow rate (<0.5 mL/min) caused by impaired PGs function disrupts the balance of the oral microenvironment, leading to the overgrowth of Candida species (such as Candida albicans). The infection rate is as high as 20%-30% in patients with severe xerostomia, and it is prone to recurrence (42).This local immune imbalance may trigger a chain reaction, increasing the risk of oral infections on one hand, and on the other hand, affecting the cellular differentiation, metabolism, and functional status of the systemic immune system through cytokine release and immune cell migration, ultimately breaking the body’s immune homeostasis mechanism. This kind of immune microenvironment change from local to systemic may not only affect the efficacy of radiotherapy, but also increase the risk of infectious complications in patients after treatment.

## 4 The impact mechanism of radiation dose and volume on the immune microenvironment of PGs

### 4.1 Changes in the number and function of immune cells

#### 4.1.1 Lymphocytes

**Figure 1:**
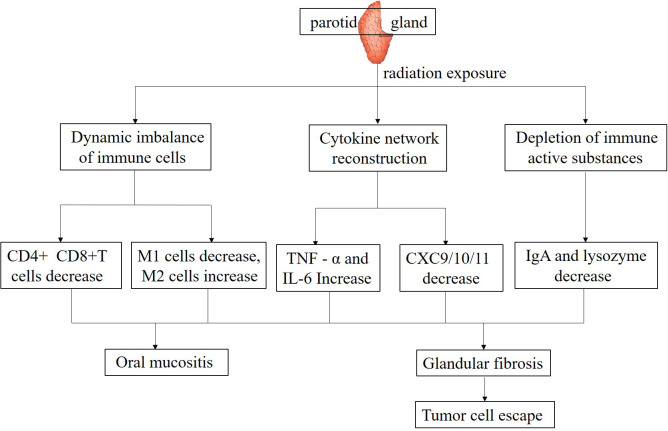
Mechanisms underlying the effects of parotid gland irradiation on the immune microenvironment.

High dose irradiation directly kills lymphocytes, inhibiting their proliferation and differentiation (44). Research has found that during radiotherapy for NPC, whether using IMRT or ART, the average radiation dose and D50 values of the PGs exhibit distinct variations, and these dosimetric parameters have been strongly correlated with the development of xerostomia. Following radiotherapy, patients demonstrate significant reductions in the neutrophil-to-lymphocyte ratio (NLR), as well as in absolute lymphocyte and neutrophil counts, with statistically significant changes indicating a notable alteration in the immune cell profile (45). CD4+ T cells serve as essential orchestrators in immune responses, playing a pivotal role in both augmenting immune activation and maintaining immunological equilibrium. A reduction in the count of these cells can lead to a significant impairment of both cellular and humoral immunity, thereby compromising the body’s overall defense mechanisms (46). CD8+T cells, as cytotoxic T cells, are crucial for killing tumor cells. The decrease in their quantity and function can affect the body’s immune surveillance and clearance ability against tumor cells (47). Studies have revealed that radiation can induce sialadenitis, leading to alterations in lymphocyte subsets within the gland. Immunohistochemical analysis demonstrated that in the irradiated submandibular gland, the inflammatory cell infiltrate predominantly consisted of CD3+ T lymphocytes and cytotoxic T cells. CD3+ T cells exhibited a distinct spatial distribution, primarily accumulating in the periacinar regions, while their presence was also noted in a scattered pattern within the peri-epithelial and intraepithelial compartments (48). Cumulative research evidence indicates a strong correlation between salivary gland fibrosis and reduced salivary secretion. This pathological process may commence as early as 8 weeks post-irradiation, underscoring the rapid onset of radiation-induced glandular damage (49). In an observation of the PGs of pigs, after receiving 15 Gy of radiotherapy, Masson’s trichrome staining analysis revealed a progressive deterioration of PG fibrosis by the 300th day post-irradiation. Concurrently, a significant upregulation of genes associated with extracellular matrix (ECM) remodeling and fibrotic processes was observed. Following the onset of glandular fibrosis, an increase in the infiltration of inflammatory cells was noted, accompanied by a marked reduction in lymphocytes, ultimately leading to a compromised immune function within the gland (50). Another clinical study reported that after the salivary glands received an irradiation dose of 66 Gy, on days 35, 80, and 105 post-irradiation, a comprehensive analysis was conducted to elucidate the association between the extent of salivary gland fibrosis and the concentrations of pro-inflammatory cytokines in saliva. The findings revealed a statistically significant positive correlation, indicating that as the radiation dose escalated, the levels of inflammatory mediators within the salivary glands correspondingly increased (51) (**Figure 1**).

#### 4.1.2 Macrophages

Macrophages have important functions in the immune microenvironment, such as phagocytosis of pathogens, antigen presentation, and secretion of cytokines (52). After irradiation of the PGs, the function of macrophages may change (53). High-dose radiation exposure can potentially attenuate the phagocytic function of macrophages, thereby compromising their capacity to eliminate pathogens and clear tumor cell debris. This impairment disrupts the integrity and homeostasis of the immune microenvironment, hindering its optimal functioning (54). On the other hand, the polarization state of macrophages may undergo changes (55). In physiological conditions, macrophages exhibit a dichotomous polarization state, differentiating into the classically activated M1 phenotype and the alternatively activated M2 phenotype (56). M1-polarized macrophages are characterized by their potent pro-inflammatory and anti-neoplastic properties, actively engaging in immune responses against pathogens and tumor cells. Conversely, M2-polarized macrophages primarily function in inflammation resolution and tissue remodeling processes. However, within the tumor microenvironment, these M2 macrophages can paradoxically facilitate tumor progression, angiogenesis, and metastatic dissemination (57). After irradiation of the PG, it may promote macrophage polarization towards M2 type, thereby altering the anti-tumor tendency of the immune microenvironment and facilitating immune escape of tumor cells (58). Investigations have demonstrated that within radiation-damaged salivary glands, an elevation in the proportion of M2 macrophages among peripheral blood mononuclear cells can mitigate sterile inflammation and foster tissue regeneration. This is achieved through the clearance of extracellular High Mobility Group Box-1 (HMGB1) and the induction of Insulin-like Growth Factor 1 (IGF1) production. These effects are likely mediated by the immunomodulatory properties of the M2-macrophage-dominant cellular fraction (59) (**Figure 1**).

### 4.2 Imbalance of cytokine network

#### 4.2.1 Pro- and anti-inflammatory cytokines

Cytokines, a family of small-molecular-weight proteins secreted by immune cells, are pivotal regulators of immune responses (60). Upon salivary gland irradiation, the cytokine network undergoes dysregulation. Evidence indicates that radiotherapy elevates the expression of pro-inflammatory cytokines, including tumor necrosis factor-α (TNF-α) and interleukin-6 (IL-6), within PG tissue (61). TNF-α initiates inflammatory cascades and facilitates the recruitment and activation of immune cells. However, persistent overexpression of TNF-α can induce hyperinflammation within the immune microenvironment, resulting in collateral damage to normal tissues (62). IL-6 not only drives inflammatory processes but also plays a crucial role in tumor cell proliferation, invasion, and metastasis (63) (**Figure 1**). Concurrently, the expression levels of anti-inflammatory cytokines, including interleukin-10 (IL-10), may be altered. IL-10 functions to suppress immune responses and mitigate inflammatory injury. Aberrant IL-10 expression can trigger immune dysregulation, failing to counterbalance pro-inflammatory cytokines adequately and thus disrupting the immune microenvironment’s homeostasis (64).

Meanwhile, ionizing radiation activates the ATM/ATR pathway by inducing DNA damage, which in turn triggers the activation of the nuclear transcription factor NF-κB (41). Excessive activation of NF-κB in PG tissue can significantly upregulate the transcriptional expression of pro-inflammatory cytokines TNF-α and IL-6. Experimental data show that its activation level is positively correlated with radiation dose: after 30 Gy irradiation, the nuclear translocation rate of NF-κB increases by 2.3 times compared with the control group, directly leading to the amplification of the inflammatory cascade reaction (9). Additionally, radiation impairs the oxidative stress defense capacity of PG cells by inhibiting the Nrf2 antioxidant pathway, thereby exacerbating immune cell apoptosis. A significant negative correlation is observed between the decreased survival rate of CD4+ T cells and the downregulated expression of Nrf2 (65).

#### 4.2.2 Chemokines

Chemokines are a type of cytokine that can attract immune cells to migrate in a targeted manner. After irradiation, the expression and secretion of chemokines in the PGs will also be affected (66). After ionizing radiation (IR) exposure of the PGs, the concentrations of CXC ligand 9 (CXCL9) and CXC ligand 11 (CXCL11) significantly declined at 2, 7, 14 days post-IR, yet recovered to baseline levels by 30 days. For CXC ligand 10 (CXCL10), significant decreases were observed at 7, 14 days post-IR, with no significant differences noted at 2 and 30 days relative to untreated controls. In contrast, CXC ligand 2 (CXCL2) levels remained significantly suppressed across all measured time points compared to controls. Under physiological conditions, chemokines guide immune cells to the tumor site for anti-tumor activity. However, radiotherapy-induced chemokine dysregulation can impede immune cell infiltration into the tumor microenvironment, attenuate the systemic anti-tumor immune response, and disrupt immune cell interactions and cooperative anti-tumor functions within the immune microenvironment (67).

Radiation can specifically downregulate the expression of Th1-type chemokines such as CXCL9 and CXCL11, with their concentrations dropping to 40%-50% of the baseline at 7 days post-irradiation, leading to reduced infiltration of CD8+ T cells and NK cells (9). Notably, CXCL10 exhibits a biphasic change after radiation: it transiently increases in the early stage (day 2) due to DNA damage stress, but continuously decreases in the late stage (day 14) due to parenchymal damage of the gland. This fluctuation directly affects the antigen-presenting function of dendritic cells (8). These findings suggest that targeted regulation of the chemokine network (such as CXCL10 agonists or CCL2 inhibitors) may serve as a potential strategy to improve the immune microenvironment of the PG (**Figure 1**).

### 4.3 Changes in secretion of immune active substances

#### 4.3.1 IgA in saliva

As mentioned earlier, the saliva secreted by the PGs contains IgA, which is an important component of mucosal immunity and can prevent the adhesion and invasion of pathogens on the surface of oral mucosa (37). After radiotherapy, PG function is impaired, saliva secretion decreases, and the content of IgA also decreases accordingly (68). The decrease in IgA levels weakens the local immune defense ability of oral mucosa, making it easier for pathogens to invade the body, thereby affecting the stability of the entire immune microenvironment (43). Furthermore, IgA likely participates in modulating immune cell activity. A decrease in IgA secretion can indirectly impair immune cell function and disrupt their interactions within the immune microenvironment (69). Radiation-induced oral mucositis represents the most prevalent acute adverse event in HNC radiotherapy. Manifesting usually in the second week of treatment, this condition persists for weeks post-radiotherapy completion, frequently leading to pain, decreased salivation, and impaired oral mucosal defense mechanisms (42) (**Figure 1**).

#### 4.3.2 Other immunologically active substances

The PG secretes additional immune-active molecules, including lysozyme and lactoferrin. Lysozyme exerts antibacterial effects by disrupting bacterial cell walls, while lactoferrin exhibits multifunctional activities, encompassing antibacterial, antiviral actions, and immune-regulatory functions (70). Radiotherapy for HNC may induce alterations in the oral microenvironment, affecting the secretion of immunoactive substances, thereby impairing the antimicrobial and antiviral capacity as well as the immune regulatory functions of the oral cavity (8). Meanwhile, changes in these immune active substances may also affect the chemotaxis, activation, and other processes of immune cells, resulting in various negative impacts on the immune microenvironment (71).

Studies have shown that lysozyme can affect the activity and function of regulatory T (Treg) cells. Under normal physiological conditions, Treg cells can effectively suppress excessive immune responses and maintain immune homeostasis. When lysozyme detects pathogen invasion, it inhibits the immunosuppressive function of Treg cells, enabling more efficient activation of immune cells such as effector T cells, thereby enhancing the body’s ability to clear pathogens. After radiotherapy for HNC, the oral microenvironment undergoes changes, leading to a reduction in lysozyme secretion. Consequently, the inhibitory function of Treg cells cannot be effectively suppressed, resulting in insufficient activation of immune cells like effector T cells, decreased oral antibacterial capacity, and imbalanced immune regulatory function. This may trigger a series of issues such as increased infection risk and persistent inflammation (**Figure 1**).

Lactoferrin is a glycoprotein with multiple physiological activities, and in addition to its antibacterial and antiviral properties, it plays a central role in immune regulation. Lactoferrin can regulate the differentiation, proliferation, and function of Treg cells by directly binding to receptors on the surface of Treg cells. In the normal oral microenvironment, this helps maintain immune tolerance and prevents excessive immune responses from damaging oral tissues. However, after radiotherapy for HNC, lactoferrin secretion decreases, leading to a lack of sufficient stimulatory signals for the differentiation and functional maintenance of Treg cells. As a result, the number and function of Treg cells decline, disrupting immune tolerance and causing an imbalance in local immune responses in the oral cavity, which may result in exacerbated inflammatory reactions and other phenomena.

In summary, lysozyme and lactoferrin interact with Treg cells through different mechanisms to jointly maintain immune system balance. Radiotherapy for HNC disrupts the secretion of these two immune-active substances in the oral microenvironment, which in turn affects Treg cells, triggering a series of negative changes in the immune system, including impaired immune defense function, imbalanced immune regulation, and abnormal inflammatory responses. A deeper understanding of the relationship between them is of great significance for developing intervention strategies to improve the oral immune microenvironment after radiotherapy.

## 5 Current status of clinical research

### 5.1 Correlation between PGs irradiation dose, volume, and immune indicators

Multiple investigations have demonstrated the time-dependent alterations in immune cell populations and cytokine concentrations within the peripheral blood of HNC patients both prior to and following radiotherapy (72, 73). During radiotherapy for HNC, a progressive decrease in peripheral blood lymphocytes is observed as the radiation dose delivered to the PGs escalates (74). This result indicates that radiation-induced PG damage not only directly disrupts the homeostasis of the local immune microenvironment and inhibits local immune functions such as mucosal immune responses, but also may exert negative effects on the proliferation, differentiation, and survival of lymphocytes in peripheral blood through systemic regulatory pathways including humoral circulation and cytokine networks. This further leads to a decrease in systemic lymphocyte counts and ultimately impairs the overall immune defense function of the organism.

**Figure 2:**
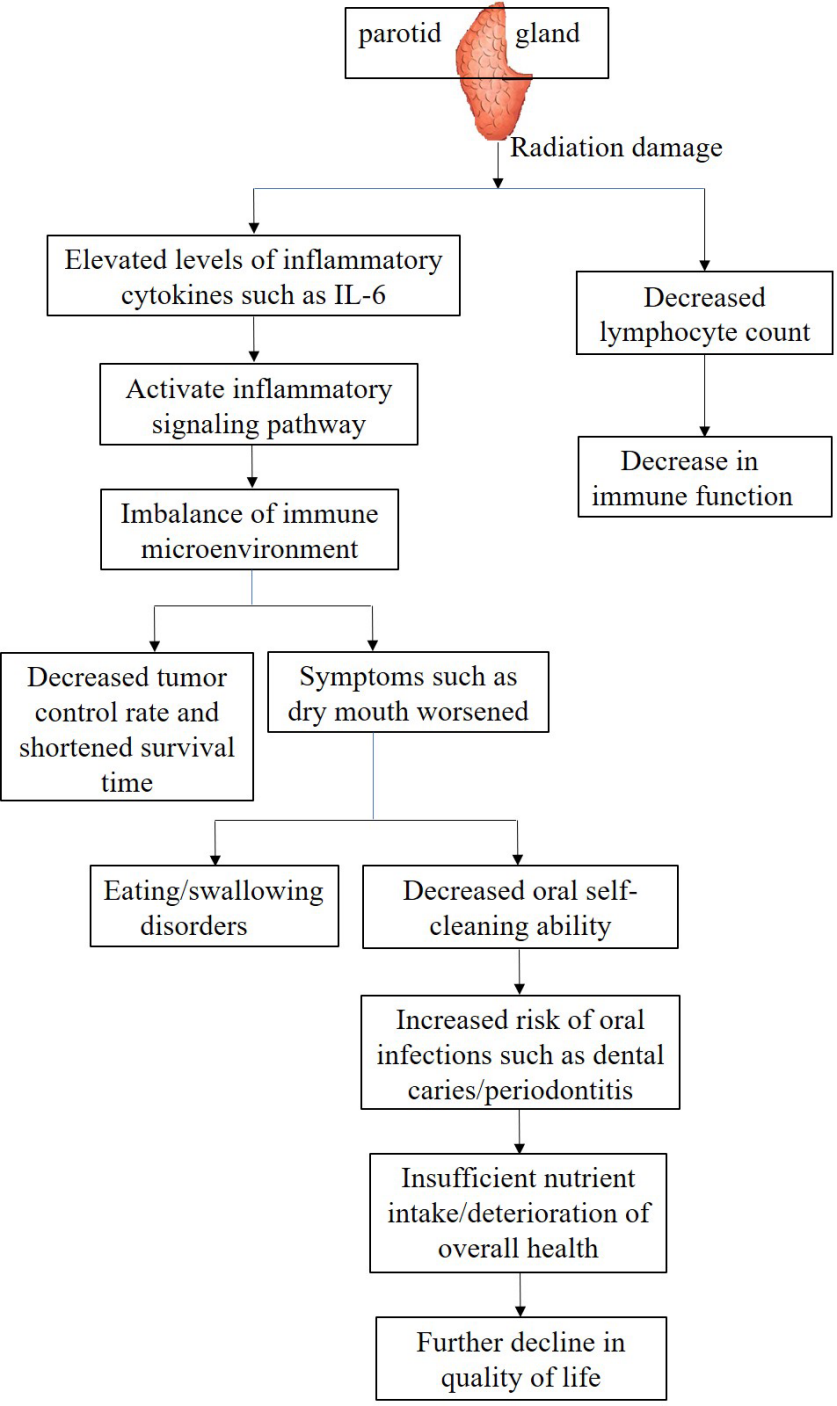
Effects of parotid gland damage on immune parameters and quality of life.

As the irradiated volume of the PG increases, local oxidative stress induced by ionizing radiation can significantly activate inflammatory signaling pathways, stimulating innate immune cells such as fibroblasts and macrophages to synthesize and release IL-6 in large quantities. The elevation of IL-6 levels can not only serve as a biological marker of the severity of radiation-induced PG injury, but also activate the Janus kinase/signal transducer and activator of transcription (JAK/STAT) pathway throughout the body through endocrine pathways. It can also disrupt the dynamic balance of the immune inflammatory network, exacerbate treatment-related adverse reactions such as radiation mucositis and fatigue. Clinical studies have shown that in patients with oropharyngeal cancer, irreversible deterioration of salivary gland secretion function occurs when the radiation dose received by the PG exceeds 20 Gy (10); The synergistic effect of mucosal barrier damage caused by salivary gland dysfunction and radiation damage to cervical lymph nodes may be an important reason for the decrease in peripheral blood lymphocytes. Further data shows that for every 10 Gy increase in the average dose to the PG, the total lymphocyte count in peripheral blood can decrease by about 15% (75). These findings suggest that the radiation dose and exposure volume of the PG are key factors affecting immune parameters, which jointly shape the immune microenvironment state through local and systemic immune regulatory networks (**Figure 2**).

### 5.2 Impact on patient prognosis and QOL

The effects of parotid irradiation dose and volume on the immune microenvironment are also reflected in patient prognosis and QOL. Research has demonstrated that IMRT offers enhanced PGs protection in NPC patients. By delivering lower radiation doses, IMRT effectively reduces the prevalence of xerostomia, whereas conventional radiotherapy techniques deliver higher radiation doses and larger irradiation volumes to the PGs, resulting in severe xerostomia, decreased tumor control rates, and shortened survival times (76). This may be attributed to impaired immune surveillance and diminished tumor cell clearance capacity due to microenvironmental damage.

From the perspective of QOL, radiation-induced xerostomia not only impairs essential daily activities like mastication and deglutition but also heightens the susceptibility to oral infections by disrupting the immune microenvironment, further compromising patient well-being. For instance, reduced salivary flow impairs oral self-cleaning, leading to dental caries, periodontitis, and other complications. These conditions exacerbate pain, impair nutritional intake, and create a vicious cycle that severely affects recovery and QOL (**Figure 2**).

## 6 Strategies to optimize radiotherapy for reducing impact on PGs and the immune microenvironment

### 6.1 Advancements in radiotherapy techniques

In the field of radiotherapy for HNC, PG protection is of crucial importance. Different radiotherapy techniques exhibit significant differences in their impact on PG radiation dose and overall immune function. Traditional 3D-CRT uses imaging technology to generate three-dimensional images of tumors and surrounding tissues, which can reduce the radiation dose received by surrounding healthy tissues to a certain extent. However, studies have shown that during 3D-CRT for HNC, the average radiation dose to the PG can reach 56.7 ± 0.7 Gy (77). This relatively high radiation dose has a substantial impact on PG function, leading to complications such as xerostomia and severely reducing patients’ QOL. Meanwhile, high-dose irradiation significantly reduces ARG1 levels in the PG, which may indicate that radiation-induced damage to macrophages in the PG could promote an M1 pro-inflammatory phenotype (78). Some studies have pointed out that high-dose irradiation of normal tissues during radiotherapy can trigger inflammatory responses (79), indirectly affecting the activity and function of immune cells. Although there are relatively few quantitative studies on the impact of 3D-CRT on overall immune function, it is speculated that 3D-CRT may have a certain degree of negative impact on overall immune function based on its high PG radiation dose and related inflammatory responses.

IMRT adjusts radiation intensity to enable better conformity of the high-dose region to the tumor shape, representing significant progress in protecting surrounding normal tissues. Compared with 3D-CRT, JO-IMRT can reduce the average PG radiation dose to 26.8 ± 0.3 Gy, the mean dose and dose to 50% PG volume were significantly lower in the IMRT-SIB than in the ConPas 3-CRT, this dose reduction significantly improves patients’ xerostomia symptoms (77, 80). From the perspective of immune function, due to the reduced radiation dose to normal tissues including the PG, radiotherapy-induced inflammatory responses are alleviated, and indirect damage to immune cells is correspondingly reduced. Studies have observed that after IMRT treatment, the activity of immune cells such as T lymphocytes and NK cells in patients recovers within a period after radiotherapy, suggesting that IMRT has advantages in immune function protection (81).

VMAT achieves precise tumor irradiation by rotating the gantry and dynamically adjusting radiation intensity and dose rate at multiple angles. Compared with nonconformal whole brain radiotherapy (NC-WBRT), VMAT significantly reduced the dose to organs at risk such as the PG. The average dose to the PG decreased from 12.8 ± 4.9 Gy to 4.4 ± 1.9 Gy, a 65% reduction in average dose. At the same time, the machine hop count (MU) of VMAT was also higher than that of NC-WBRT (719 vs 350), shortening the treatment time (82). Franzese et al. used IMRT and VMAT for the treatment of oropharyngeal cancer and found that VMAT reduced the incidence of mucositis and dysphagia, indicating that VMAT provides better protection for normal tissues than IMRT (83). Lower PG radiation doses help alleviate complications such as xerostomia, thereby improving patients’ QOL. VMAT reduces unnecessary irradiation of normal tissues through more precise targeting, lowering radiotherapy-induced systemic inflammatory responses.

Proton therapy utilizes the unique Bragg peak phenomenon to precisely deposit high-dose radiation within tumors, significantly reducing radiation exposure to surrounding normal tissues, including the PG. In the radiotherapy of HNC such as tonsil cancer, PG cancer, and submandibular gland cancer, proton radiotherapy can significantly reduce the average dose to the contralateral PG and the V10 Gy dose (84) (**Table 1**). From the perspective of immune function, although there is no direct research confirming that proton therapy can protect patients’ immune function, proton radiotherapy provides better protection for normal tissues, reducing normal tissue damage and thereby lowering immune suppression factors induced by such damage. Clinical studies have followed up and found that immune function-related indicators such as cytokine levels and immune cell activity remain relatively stable in HNC patients after proton radiotherapy, suggesting that proton radiotherapy has a favorable protective effect on overall immune function (85).

**Table 1:** Dosimetric comparison of the contralateral parotid gland between IMPT and VMAT.

Structure	Parameter	IMPT	VMAT
Contralateral parotid	Mean (Gy)	0.1	8.8
	V10 Gy (%)	0.0	24.9
	V20 Gy (%)	0.0	0.0

In summary, for HNC radiotherapy, compared with 3D-CRT, IMRT, VMAT, and proton therapy all have significant advantages in reducing PG radiation dose. Among them, proton therapy is particularly prominent in reducing PG radiation dose and protecting overall immune function, while IMRT and VMAT also improve PG protection and reduce impacts on immune function to varying degrees. Clinicians can comprehensively consider and select the most appropriate radiotherapy technique based on specific patient conditions, such as tumor type, location, and patient physical status.

### 6.2 Adaptive radiotherapy or CCRT induced-changes of PG

The dynamic changes in the volume and position of PG during radiotherapy for HNC cannot be ignored. Multiple studies have tracked changes in PG during radiotherapy using imaging techniques such as CT. In a study targeting NPC, weekly magnetic resonance imaging(MRI) monitoring revealed that the volume of PG continued to shrink during radiotherapy, with the ipsilateral PG shrinking at a rate of 3.7 ± 3.3% per week, significantly faster on the ipsilateral side than on the contralateral side (86). Another prospective study conducted a series of CT scans of PG during radiotherapy in 13 patients with HNC. The results showed that from baseline to the 6th week of radiotherapy, the average volume of PG decreased by 37.3% (87).

In view of this, timely adjustment of radiotherapy treatment plan is of great significance. Adaptive radiotherapy (ART) technology can guide patients to understand anatomical and physiological changes, tumor target areas, and changes in the morphology and location of PG through imaging, and modify treatment plans. A study targeting patients with HNSCC evaluated the anatomical changes in the target area and PG using daily cone beam CT (CBCT) image-guided and registration techniques. Repositioning CT scans and re-planning were performed on patients at the 10th and 22nd radiotherapy sessions. It was found that as radiotherapy progressed, the volume of the target area and bilateral PGs gradually decreased, and the re-planned bilateral parotid irradiation dose was significantly reduced compared to before radiotherapy (88). This further confirms that in radiotherapy for HNC, solid radiotherapy can effectively reduce the irradiation volume of PG and lower the radiation dose to PG.

CCRT is widely used as an efficient comprehensive treatment for HNC. Numerous studies have shown that CCRT can significantly reduce tumor volume in the treatment of HNC, creating more favorable conditions for subsequent treatment (89). However, CCRT can exacerbate the toxic side effects of treatment. Research has shown that the probability model parameter TD50 for normal tissue complications during CCRT is 32.2 Gy at 4 weeks and 32.1 Gy at 6 months, while the radiotherapy alone group has 41.1 Gy at 4 weeks and 39.6 Gy at 6 months. This suggests that the tolerance dose of TD50 in the CCRT group is 7 to 8 Gy lower than that of radiotherapy alone, and in this study, it was found that the CCRT group often has a higher possibility of causing damage to parotid gland tissue (90).

In summary, during the radiotherapy process for HNC, CCRT effectively shrink the tumor due to their synergistic anti-tumor properties, laying a solid foundation for treatment. Meanwhile, ART relies on its precise irradiation technology to reduce the irradiation of PG volume, effectively achieving the goal of protecting PG and greatly improving the treatment effect and QOL. If these two treatment methods are organically combined, it can bring longer survival and better QOL of HNC.

### 6.3 Different fractionated radiotherapy induced changes of PG

In the field of radiotherapy for HNC, conventional fractionated radiotherapy (CFR) is the most traditional mode. This fractionation strategy is theoretically rooted in classical radiobiology, aiming to deliver sufficient tumoricidal doses while ensuring normal tissues have adequate time to repair radiation-induced sublethal damage. There have been many studies on the damage to the PG and the symptoms of xerostomia caused by CFR, but there is paucity of studies investigating whether hypofractionation, hyperfractionation, continuous accelerated hyperfractionation, and other fractionation regimens induce similar PG injuries.

Hypofractionated employs fewer treatment sessions with larger doses per fraction. Its advantage lies in the ability to significantly shorten the total treatment time, theoretically, it may reduce the phenomenon of tumor cell proliferation. However, for the PG, hypofractionated radiotherapy has duality. On the one hand, due to the increase in single dose fractionated, the PG receives a significantly higher dose during each irradiation, which undoubtedly increases the risk of acute and late toxicity reactions in normal tissues. On the other hand, if advanced radiotherapy techniques such as IMRT are combined with the implementation of hypofractionated radiotherapy, and the dose distribution is optimized and adjusted, effective protection of the PG can be achieved while reducing the total dose, which can reduce the occurrence of complications. Shuryak et al. analyzed 16 randomized clinical studies on radiotherapy for HNC and found that compared with CFR, optimized hypofractionated radiotherapy not only improves tumor control rate and shortens treatment time, but also reduces complications in late-reacting tissues, making it a very promising treatment method (91). Price et al. used different fractionated methods (50 Gy/20 fractions/4 weeks, 55 Gy/25 fractions/S weeks, or 54 Gy/36 fractions/l2 days (CHART)) to irradiate the parotid and submandibular glands of monkeys, and found that the number of serous acini decreased in all three groups. The CHART group had fewer serous acini occupying the volume of the PG, suggesting that accelerated hyperfractionated radiotherapy may be more likely to damage the PG (92). Wu et al. explored a study on the treatment of radiation-induced parotid dysfunction in NPC using different fractionated methods. They found that the proportion of patients in the late course accelerated hyperfractionation radiotherapy group who developed acute parotitis was significantly higher than that in the CFR and the IMRT, and the incidence of oral ulcers was also higher than the two groups. This may be due to the increased radiation dose to tissues in the short term of continuous accelerated hyperfractionation therapy, which aggravated the acute radiation reaction of normal tissues (93). Multiple studies suggest that different fractionated methods for treating HNC can indeed cause damage to the PG, leading to a decrease in salivary gland secretion function and indirectly affecting the patient’s immune function.

Therefore, in clinical practice, it is necessary to comprehensively consider multiple factors such as tumor location, precision of radiotherapy techniques, and individual differences of patients, weigh the advantages and disadvantages of different fractionated radiotherapy, in order to achieve optimal protection of the PG and effective control of the tumor. In the future, with the continuous development of radiotherapy technology and in-depth research on radiobiological mechanisms, it is expected to further optimize the fractionated radiotherapy plan, better balance the relationship between tumor treatment effectiveness and PG protection, and bring better treatment experience and QOL of HNC.

## 7 Summary and perspectives

In the process of radiotherapy for HNC, the negative impact of PG damage on patients’ immune function has gradually attracted attention. Current studies have clearly demonstrated that after radiation exposure to the PG, changes in radiation dose and volume alter the composition and distribution of immune cell populations, disrupt cytokine balance, and affect immunologically active secretions. These changes ultimately impair patient prognosis and QOL. Although existing studies have revealed some relevant effect patterns—such as how different radiotherapy fractionation regimens cause varying degrees of PG damage, which indirectly affects immune function—numerous research directions remain to be explored.

Future researches should focus on elucidating the molecular mechanisms linking PG irradiation to immune dysregulation. It is currently known that radiotherapy induces tumor cells and normal tissue cells to release various cytokines and chemokines, which may act as bridges between PG damage and immune function changes. For example, studies have found that radiotherapy can induce tumor cells to release damage-associated molecular patterns (DAMPs), which activate innate immune responses. However, in the context of PG damage, how DAMPs regulate immune cell infiltration into the PG and surrounding tissues, as well as their impact on systemic immune cell function, remains incompletely understood. In-depth exploration of these molecular mechanisms will lay the foundation for developing targeted interventions, with the potential to mitigate radiotherapy-induced damage to the PG and immune function by regulating key molecular pathways.

Improving radiotherapy technology represents another important future research direction. With continuous technological advancements, novel radiotherapy techniques such as proton therapy and heavy ion therapy have gradually entered clinical practice. Proton therapy, due to its unique Bragg peak characteristic, can precisely deliver energy to the tumor target volume while significantly reducing scattered doses to surrounding normal tissues, including the PG. Future research should conduct large-scale clinical studies to compare the efficacy of different radiotherapy technologies in reducing PG radiation dose and preserving immune function. Optimizing radiotherapy planning and improving precision will be crucial for minimizing damage to the PG and immune microenvironment.

Optimizing fractionation regimens is equally vital. Current research on how different fractionation approaches (such as hypofractionation, hyperfractionation, and continuous accelerated hyperfractionation) affect PG damage and immune function remains insufficiently thorough. Hypofractionated radiotherapy delivers larger single-fraction doses, which theoretically shortens treatment duration and reduces tumor cell repopulation but may increase the risk of acute and late toxicities in normal tissues. In contrast, hyperfractionation, which increases the number of fractions while reducing single-fraction doses, theoretically facilitates normal tissue repair, but its protective effect on PG immune function in clinical practice requires further validation. Future studies should conduct more prospective, multicenter randomized controlled trials to explore the relationship between PG damage and immune function changes under different fractionation regimens, aiming to identify optimal radiotherapy fractionation patterns that maximize protection of PG function and its mediated immune function while ensuring effective tumor control.

Advancing multidisciplinary approaches, such as combining radiotherapy with immunotherapy, also holds broad research prospects. Immunotherapy has achieved significant progress in treating various tumors; combining it with radiotherapy is expected to synergistically enhance anti-tumor immune responses while reducing radiotherapy-induced damage to normal tissues, including the PG. On one hand, radiotherapy can induce tumor cell antigen release and activate anti-tumor immune responses, which can be enhanced by immunotherapy to improve tumor control rates. On the other hand, rational design of combined treatment regimens may regulate the immune microenvironment and alleviate radiotherapy-induced immune damage to normal tissues like the PG. Future research should investigate the optimal timing, dosage, and modalities for combining radiotherapy with different immunotherapeutic approaches (such as immune checkpoint inhibitors and adoptive cellular immunotherapy), exploring their impact on parotid immune function to provide more effective comprehensive treatment strategies for HNC.

Research on immune function impairment caused by PG damage during HNC radiotherapy is still in its developmental stage. Through in-depth studies of molecular mechanisms, improvements in radiotherapy technology, optimization of fractionation regimens, and advancement of multidisciplinary combination therapies, it is expected that future efforts will maximize reduction of damage to the PG and immune microenvironment, ultimately improving treatment outcomes and patient well-being of HNC.
